# Comparison of canine owner profile according to food choice: an online preliminary survey in France

**DOI:** 10.1186/s12917-022-03258-9

**Published:** 2022-05-04

**Authors:** S. Hoummady, M. Fantinati, D. Maso, A. Bynens, D. Banuls, N. R. Santos, M. Roche, N. Priymenko

**Affiliations:** 1 Université Paris-Est, Ecole Nationale Vétérinaire d’Alfort, UMES, 7 avenue du Général de Gaulle, 94704 Maisons-Alfort, France; 2 École Nationale Vétérinaire de Toulouse, 23 chemin des Capelles, 31300 Toulouse, France; 3 Université Paris-Est, Ecole Nationale Vétérinaire d’Alfort, CHUVA, 7 avenue du Général de Gaulle, 94704 Maisons-Alfort, France; 4 FACCO, 46 Boulevard de Magenta, 75010 Paris, France; 5 TOXALIM, Université de Toulouse, Institut National de La Recherche Agronomique (INRA), École Nationale Vétérinaire de Toulouse (ENVT), BP 87614, 23 chemin des Capelles, 31076 Toulouse cedex, France

**Keywords:** Pet food, Survey, Non-conventional diets, Canine nutrition, Biologically Appropriate Raw Food

## Abstract

**Background:**

Nowadays, more people are treating dogs as family members. This reflects their increased attention towards their nutrition, with renewed interest for non-conventional diets such as Biologically Appropriate Raw Food/ Bones and Raw Food in United States (BARF) or homemade. In previous studies, owners feeding their dog non-conventional diets reported lower levels of trust in veterinary advice. The aim of the study was to identify differences in lifestyle between owners feeding dogs non-conventional diets and those feeding conventional diets (i.e., dry/wet pet food) to give further insight for improving communication between veterinarians and owners.

**Results:**

A total of 426 surveys were usable. Fifteen percent of the participants lived in the metropole of Paris and had more than one dog (mean 1.72 dogs). Thirty-eight percent of the survey respondents stated that their dogs were fed exclusively with non-conventional diets, while 55% declared using conventional diets alone (not considering treats). The study canine population was for the most part neutered (63%) and purebred (68%). Amongst owners feeding conventional diets exclusively, 47% determined how much food to feed by consulting the feeding guidelines on the packaging, and only 28% said that the amount of food was prescribed by their veterinarian or veterinary nurse. Out of the participants feeding non-conventional diets, 65% declared that the information for formulating the recipes was gathered on the internet or in non-veterinary books. When compared with owners feeding exclusively conventional diets, those feeding non-conventional diets were living more frequently outside the metropole of Paris, had fewer children (0.23 ± 0.57 vs 0.37 ± 0.78; *p* = 0.03) and had more frequently other animals. They also dewormed less often their pets, walked their dog more each day (91 vs 78%; *p* < 0.001) and without leash for more than 6 h per week (46 vs 31%; *p* = 0.003).

**Conclusions:**

This survey described differences in the habits of owners feeding dogs non-conventional diets in comparison with those feeding conventional diets. Data suggest that owners using non-conventional diets may be more attentive to the ethological needs of their dog which could be a starting point for practitioners for achieving better client-veterinarian communication.

**Supplementary Information:**

The online version contains supplementary material available at 10.1186/s12917-022-03258-9.

## Background

The dog population in France was evaluated to be 7.6 million in 2018 with a 4% increase compared with 2016 [8]. These numbers represent a growing market for the pet food industry which invests continuously in new products trying to meet the preferences of pet-owners. The latter’s expectations have evolved rapidly in recent years (e.g., novel vision of “natural” dog food, increase in Biologically Appropriate Raw Food/prey model diets). In this context, to attract consumers, marketing professionals are more and more interested in the owners’ perception [3]. Some marketing ploys may bias the owner’s perception of the nutritional quality of the chosen diet: a recent study about the Italian pet food buyers, reported that the presence of “natural” ingredients was considered as an important indicator of pet food quality from pet owners point of view [21]. On the other hand, there is an increasing interest of owners about nutrition trends like “grain free”, “homemade”, “raw food” or “vegetarian” diets for dogs. According to a survey study in English-speaking countries (Australia, Canada, New Zealand, UK and USA) concerning canine feeding practices by owners between 2008 and 2018, the proportion of dogs fed with inclusion of non-conventional diets like home-made diets or vegetarian appears to be increasing [6]. These changes in feeding practices are raising concerns about microbiological risks regarding owners and dogs when raw products are involved [19]. Furthermore, analysis of these recipes frequently showed several nutrients below recommendations [17] which can be extremely dangerous for some pets (Kitten, puppy, senior animals, cardiac dogs). Recently grain free diets has been linked to cardiac disease [1]. Pet nutrition is the centre of owner preoccupation and veterinarian face hardly to multiple question between science and marketing. Because the aforementioned study by Dodd et al. [6] showed different results from one country to another, conducting surveys in different countries will be a useful tool to improve veterinary education in the field of nutrition. In absence of information from veterinarians, owners will search on the internet [13, 14]. Communication is a cornerstone of nutrition consultation but the profile of owners feeding dogs non-conventional diets (NCD) compared to profile of owners feeding dogs with conventional diets (CD) (i.e., dry/wet pet food) has not been clearly defined and is essential for good veterinarian-client communication. A marketing study has defined 3 profiles of owners with regards to the relation dog–human and anthropomorphism items, “Dog people”/ “Dog parents”/ “Dog owners”, [3]. But these definitions do not give information on potential differences in terms of lifestyle (canine and human) between owners feeding dogs non-conventional diet like homemade diets (NCD) versus those feeding conventional diets (CD) like industrial diets. Another study reported some characteristics of pet owners who prefer to feed dogs with raw animal products, like majority women, aged around 41 years-old and mainly without children [14]. Current studies mainly focus on owners’ perceptions and motivations, but only few about life habits.

In France, only one nutrition epidemiological survey [4] was conducted and concerned canine obesity:14.1% of dogs were fed only with a home-prepared diet but few details were given about pet owners feeding a NCD.

The aim of the present study was to identify potential differences in terms of lifestyle and habits between dog owners feeding a NCD and those feeding a CD in the French population. The objective is to increase the knowledge of veterinarians to better address this evolving situation. Moreover, this study was an opportunity to assess the French online dog-owners’ population. To this end, an online survey was conducted during the COVID-19 lockdown at the beginning of 2020.

Authors hypothesized that dog owners using NCD are older than those using CD, and that the number of children is lower in the population of owners feeding NCD as observed in Morgan et al. [14] survey in US. According to the model of “the wolf”, frequently used by marketing, there is the assumption that dogs of owners using NCD are more frequently males, entire and purebred [13]. Finally, we supposed that dogs of owners using NCD lived more frequently in a household with several animals, as wild canids do in their natural environment. Finally, due to the lack of trust in veterinarians reported in other studies, supposed that owners using NCD are deworming less their animals.

## Results

### Survey participants

The survey was stopped with 561 answers. After cleaning the data base, 429 dog owners remained (Table 1). Fifteen percent of owners resided in the metropole of Paris (65/429), 42% aged 26–40 years old (179/429) and 49% had a household of 2 people (209/429). In the sample, the average number of dogs per family was 1.72 (SD: ± 1.17; range 1–8). The diet choices were quite variable: 38% (162/429) of owners declared feeding only a NCD (BARF, prey model, whole prey, cooked homemade food)), 55% (235/429) stated an exclusive CD and 7% (29/429) used a mix of both (NCD and CD).

**Table 1:** Demographics of survey respondents (*n* = 429)

	Dog owners n (%)
Region	
Out of Paris area	361 (85%)
Metropole of Paris	65 (15%)
Number of children	
Mean ± SD	0.33 ± 0.72
Median (Range)	0 (0—6)
Age	
18–25 years old	126 (30%)
26–40 years old	179 (42%)
41–60 years old	103 (24%)
> 60 years old	18 (4%)
Household	
1 people	81 (19%)
2 people	209 (49%)
3 or more	136 (32%)
Number of dogs	
Mean ± SD	1.73 ± 1.16
Median	1 (1–8)
Type of diets	
Conventional (dry and/or wet pet food) exclusively (CD)	235 (55%)
Non-conventional exclusively (homemade, BARF, …) (NCD)	162 (38%)
Mix of both (CD and NCD)	29 (7%)

### Canine population

Forty-eight percent of dogs in this study were females (203/429; Table 2). Mean age was 4.45 years (SD: ± 3.15). Most dogs were neutered (64%; 268/429; Table 2). Mean weight was 22.18 kg (SD: ± 11.60) and 68% of dogs were purebred. Environment and lifestyle were varied. Thirty-eight percent (163/429) of dogs practiced a sport activity (i.e. agility, …). Sixty-four percent (274/429) lived in a house in contrast with apartment or other situations (i.e.: kennel, apartment, and a house). Forty-two percent (174/429) had daily walks lasting 1 to 2 h in total. More than half (53%; 226/429; Table 2) had the possibility to go outdoor several times per day. Sixty-nine percent (296/429) lived in the same household with other animals (dogs, cats, …) and 77% (326/429) had toys and used them on their own.

**Table 2:** Characteristics of dogs enrolled in the survey

	DOGS n (%)
Gender	
Female	203 (48%)
Male	223 (52%)
Age (years)	
Mean ± SD	4.45 ± 3.15
Median (Range)	4 (1–20)
Neutered	
Yes	268 (63%)
No	158 (37)
Weight (kg)	
Mean ± SD	22.18 ± 11.60
Median (range)	21.25 (1.96–62)
Body condition according to owner	
Normal	356 (84%)
Slightly overweight	49 (12%)
Slightly underweight	19 (5%)
Obese	1 (< 1%)
Underweight	1 (< 1%)
Purebred	
Yes	290 (68%)
No	136 (32%)
Muscular mass according to owner	
Normal and muscular	370 (87%)
Low	56 (13%)
Sport activity	
Yes	163 (38%)
No	263 (62%)
Habitat	
Apartment	148 (35%)
House	274 (64%)
Other (both, outside, …)	4 (1%)
Time per day spent outside by the dog	
Less than 30 min	15 (4%)
30—60 min	89 (21%)
1 -2 h	174 (41%)
More than 2 h	148 (35%)
Frequency of walk with the dog (outside garden or house)	
Occasionally (the week-end, …)	60 (14%)
Rarely (during holidays, …)	9 (2%)
Each day, multiple times	226 (53%)
Each day, one time	131 (31%)
Contact with other animals at home	
Yes	296 (69%)
No	130 (31%)
Toys	
Yes, but not used	81 (19%)
Yes, used	326 (77%)
No	19 (5%)

### Dogs’ feeding habits practiced by owners using conventional diets (CD)

Forty-four percent (104/ 235) of CD were veterinary brands (in France, by market share: Royal Canin^©^, Hill’s^©^, Virbac HPM^©^, Purina Pro Plan^©^, Specific^©^). Only for 28% (66/235) the food amount was prescribed by a veterinarian (or a nurse). Seventy-three percent (172/235; Table 3) of dogs had two meals or more per day.

**Table 3:** Habits of owners using CD

	Owners using CD – 235 individuals n (%)
Veterinarian brand	
Yes	104 (44%)
No	131 (56%)
Amount	
Prescribed by the veterinarian or nurse	66 (28%)
As indicated on the package (feeding guidelines)	105 (47%)
Prescribed by someone else (not a veterinarian, a nurse, or a manufacturer)	35 (15%)
Ab libitum	29 (12%)
Number of meals/days	
Ab libitum	19 (8%)
Once a day	44 (19%)
2 or more	172 (73%)

### Dogs’ feeding habits practiced by owners using non-conventional diets (NCD)

Eighty-five percent of recipes were BARF/Whole prey rations. Sixty-six percent (106/162; Table 4) of recipes came from online sources. Veterinarian recipes corresponded to 8% of rations. Seventy-two percent (116/162) of NCD did not have vitamin and/or mineral supplements.

**Table 4:** Habits of owners

	Owners using NCD – 162 individuals n (%)
Type of recipe	
BARF/Whole prey…	137 (85%)
Cooked homemade	25 (15%)
Number of meals/days	
Once a day	36 (22%)
2 or more	126 (78%)
Origin of recipe	
Prescribed by a veterinarian during a consultation	4 (2%)
Prescribed by a veterinarian on the internet	10 (6%)
Personal recipe	28 (17%)
Recipe from a book written by a veterinarian	14 (9%)
Recipe from the internet or a book but not calculated by a veterinarian	106 (66%)
Presence of vitamin and/or mineral supplements	
Yes	46 (28%)
No	116 (72%)

### Comparison of owners using NCD versus CD

Owners using NCD, compared to those using exclusively CD, lived less in the metropole of Paris (9 vs 20%; *p* = 0.007; Table 5), had fewer children (*p* = 0.03) and a smaller household (*p* = 0.004). They had more frequently other animals at home (80 vs 63%; *p* < 0.001; Table 5) and allowed more daily outdoor access to their dogs (91 vs 78%; *p* < 0.001; Table 5). They also tended to treat less for internal parasites, in fact, significantly fewer of them dewormed their dogs more than once a year (62 vs 89%; *p* < 0.0001; Table 5). Pet-owners feeding NCD walking more their dogs without leash 6 h/week than owners feeding CD (46 vs 31%; *p* = 0.003; Table 5). Among NCD 50 dogs were mix-breed and the most common breeds were Belgian Shepherd Malinois (*n* = 5/162), German Shepherd (*n* = 4/162); White Shepherd (*n* = 4/162); Golden Retriever (*n* = 4/162) and Jack Russell (*n* = 4/162). Among CD, most of dogs were also mix-breed (*n* = 80/235). The most common breeds were Australian Shepherd (*n* = 15/235); Golden Retriever (*n* = 9/235); Husky (*n* = 7/235); Jack Russel (*n* = 7/235).

**Table 5:** Comparison of lifestyle between owners using NCD and owners using CD

	OWNERS USING NCD – 162 INDIVIDUALS N (%)	OWNERS USING CD – 235 INDIVIDUALS N (%)	*P*-VALUE
Region			
Paris metropole	15 (9%)	46 (20%)	0.007*
Out of Paris metropole	147 (91%)	189 (80%)	
Number of children			
Mean ± SD	0.23 ± 0.57	0.37 ± 0.78	0.03*
Aged of 40 years and older			
Yes	37 (23%)	69 (29%)	0.18
No	125 (77%)	166 (70%)	
Household (n of people)			
Mean ± SD	2.17 ± 0.90	2.49 ± 1.35	0.004*
Gender			
Female	72 (44%)	119 (51%)	
Male	90 (56%)	116 (49%)	0.27
Age (years)			
Mean ± SD	4.41 ± 2.86	4.46 ± 3.34	0.86
Neutered			
Yes	102 (63%)	153 (65%)	0.74
No	60 (37%)	82 (35%)	
Purebred			
Yes	112 (69%)	155 (66%)	0.58
No	50 (31%)	80 (34%)	
Sport activity			
Yes	68 (42%)	83 (35%)	0.21
No	94 (58%)	152 (65%)	
Time per day spent outside by the dog			
< 30 min	6 (4%)	7 (3%)	0.68
30–60 min	34 (21%)	49 (21%)	
1–2 h	71 (44%)	92 (39%)	
> 2 h	51 (31%)	87 (37%)	
Dog walked daily (outside garden or house)			
Yes	147 (91%)	183 (78%)	< 0.0001*
No	15 (9%)	52 (32%)	
Deworming more than once a year			
Yes	101 (62%)	209 (89%)	< 0.0001*
No	61 (38%)	26 (11%)	
More than 6 h of walk without a leash/week			
Yes	75 (46%)	73 (31%)	0.0003*
No	87 (53%)	162 (69%)	

Significative *p*-value were presented with the symbol *

## Discussion

The present study is the first to compare lifestyles of owners and dogs according to the dog’s diet in France. Moreover, there are very few data about the habits of French pet owners in terms of owner food choice for their dogs [4]. Last, but not least, this survey is focused on the Internet population, which is a highly active population as far as BARF and raw diets are concerned but still under-researched. Information collected in this study highlights differences between owners/dogs using NCD versus owners using CD. Owners using NCD lived more frequently outside of the metropole of Paris, had fewer children, and dewormed less than owners using CD. These results are consistent with the analysis by Morgan et al., [14], where pet owners who fed raw animal products were in majority without child (61%) and only 28% lived in an urban area. But in contrast to Morgan et al. [14] results, owners using NCD were mostly 40 years or younger. This difference can be explained by the earlier presence of the trend of raw diets and homemade food in the US compared with France or an age population more present on internet (40 years or younger). This difference must be more studied in future survey. The present study results draw a profile of a home in a residential setting, less urban, and a family more focused on “nature”. It would be interesting to explore the compliance of these owners with veterinary counselling or dog vaccination. It may well be those owners using NCD vaccinate less their dogs due to lack of veterinary trust, as observed in Morgan studies, with a tendency of pet owners feeding raw products less likely to vaccinate and deworm. This assumption agrees with the origin of the recipes used by owners using NCD. In our study, only 14 owners using NCD (9%) reported a veterinary recipe prescribed for the dog, 14 owners used a NCD recipe found in a veterinary book and the majority (83%) used recipes from the Internet or non-veterinary books, or personal prescription. Another online survey reported similar results, with only 14% of the interviewed people having asked a veterinarian or a nutrition-trained expert for advice for raw meat-based diets [13]. For these owners, veterinarians were not the first source of information about nutrition, which confirms the important role of other sources of information like the Internet [14]. When compared with owners using CD, the first source of information about food quantity was the manufacturer (47%), and veterinarians were the second (28%). This proportion of owners using veterinarian information, even if higher for owners using CD, is still low and in accordance with observations of other surveys [10, 12, 14]. There is a need of increased veterinarian communication about nutrition as suggested in a recent publication [7], especially on the Internet, where owners are searching information. This naturally suggests a requirement for more nutrition training in veterinary schools to prepare students [2] and a better vet communication about their ability in canine nutrition. This lack of trust regarding veterinarians may also have implications for animal and public health. Indeed, raw homemade food are more and more present on the market and this dietary practice is known to be associated with microbiological risks both for pets and their owners [5, 19]. Studies have reported mineral deficiencies in home-prepared diets, mainly calcium (Dillitzer et al., 2011; [18]. The Dillitzer study reported in 2011 that 60% of bone and raw food ration had major nutrient imbalances. The present study’s results confirm this danger, with only 28% of the French online respondents feeding NCD already using a mineral and/or vitamin supplement to balance their recipe. The improvement of communication regarding nutrition between veterinarians and owners using NCD may be a benefit to dogs’ health with an appropriate modification of the NCD if imbalanced.

In order to improve communication, there is a need to better understand the audience (NCD owners in the present case). This survey helps to better define the characteristics of NCD French dog owners. Compared to owners using CD, they had more frequently other animals at home, provided more often daily access to the outside to their dogs (outside of the garden and the house), and walked them more frequently off-leash for more than 6 h per week (which may be associated with a more rural lifestyle). These life-conditions provide an enriched environment for dogs [22] and closer to the species’ ethological needs. These observations may relate with the fact that the majority of owners using NCD lived outside the metropole of Paris, but even in an urban environment, there is possibility to walk a dog each day. Another hypothesis is the new trend to “natural foods”, which takes inspiration from the human food marketing [15]. According to Moscato and Machin [15], in human marketing the term “natural” is associated with authenticity, and with the idea of being a good mother. The “natural” adjective may help to simplify food decision [11, 15] by luring consumers into purchasing the idea of some health-giving properties. The trend “back to nature” is also present in pet foods, with an increased demand for this sector and a market corresponding to 25% of the total value of the pet food market in the US in 2016 [20]. This can be explained by the humanization of pets and the fact that owners transposed their own dietary choice for “natural food” on their dogs. Two common reasons for choosing raw diets are their perception as “more natural” and “healthier” [14]. In terms of communication, dogs are often compared to wolves as model of wild canids eating natural food. The comparison between dogs and wolves is very present in the online community, which may explain a choice of dog breeds with higher body weight for owners using NCD. The major argument is that, since wolves are dogs’ ancestors, food found in wild conditions by the former is supposedly optimal for a dog. This frequent comparison may have led owners using NCD to take care of ethological needs (more off-leash walk, more often daily access to the outside) of their dogs more carefully than owners using CD because of comparison with wolf lifestyle (in group, living outside, …). To explore this hypothesis, it will be mandatory to compare the ethological knowledge of owners using NCD versus owners using CD. Ethology could be a promising approach angle to discuss nutrition with owners using NCD rather than focusing only on canine dietary requirements if this hypothesis is confirmed.

Although expected, lower neutered prevalence in the NCD population compared to the CD population as presented by Morelli et al. [13] was not evidenced in our data.

The present study was centered on the online population, which is a highly active community about canine nutrition and one of the main source of information for owners [14]. Due to the social media recruitment, the high prevalence of owners using NCD in this survey is not representative of the owners using NCD in the French population as NCD owners may be more active and present on internet compared to CD owners. Nonetheless, the objective of this study was not to quantify the prevalence of owners using NCD in France. Moreover, there is no reason to believe that owners who have access to social media have a different lifestyle compared to owners who are not social media users.

The definition of “non-conventional” diets has no consensus yet [16]. The term “alternative” could be used as suggested by Parr and Remillard [16], but this expression included the trend of “grain free” and” vegetarian” kibbles in France. In the present study, “non-conventional” diets referred to “raw, homemade, vegetarian” as suggested by the WSAVA Nutritional assessment guidelines [24]. As no vegetarian diet was reported in the survey, “non-conventional” diets only included “raw” and “homemade” diets. The distinction “commercial” versus “non-commercial” was not appropriate as some new raw recipes are industrially made. A comparison between owners using raw products and owners using cooked products should be conducted to explore the profile of NCD owners and adapt communication. The low percentage of owners using cooked products in this study did not allow such comparison. The body score index was not included in the survey, due to the difficulty for owners to correctly answer the question on a internet survey. Images of the dog were requested but only few owners sent quality pictures to assess the body condition. This study was not designed to assess differences in body score index according to diet choice, but difference of format and body score should be included in a future survey.

These results led to questions about differences between owners using NCD and owners using CD, like the reason of their choices, their economic and social status and their personality profiles which can influence food choice [9]. Moreover, this study did not compare owners who used a mix of NCD and CD by lack of individuals in this group (7%). It would be interesting to explore their profiles compared to owners using NCD and CD. Additional studies are needed to explore the differences of lifestyle and personality of owners using NCD versus owners using CD in the social media population and general population visiting veterinary clinics.

## Conclusion

This study is the first to assess the differences between owners using NCD and CD in the sampled online French population. Results showed that the majority of owners feeding NCD lived outside the metropole of Paris, had fewer children but more animals at home, dewormed less frequently their dogs, had dogs with higher body weight, took them more often on a walk and left dogs off-leash more than 6 h per week. Moreover, this study shows that veterinarians are rarely consulted as a source of advice by owners using NCD, which may indicate a lack of trust in French veterinarians on the importance of pet nutrition or a lack of veterinarian’s communication regarding their nutrition competencies. These results may help to better understand these populations of owners and improve communication with veterinarians about nutrition.

## Methods

### Survey design and recruitment

A web-based questionnaire was created in French language on the Google Forms platform to recruit owners. No approval by an institutional review board was required because enrolment was on a voluntary basis. The survey was anonymous, and a question asked the authorisation to use data for publication. No animal has been used in this protocol.

The survey was beta-tested among authors. The survey consists of 103 questions and was inspired by the questionnaire or [4]. Twenty-nine questions were mandatory, moreover, there were 30 open-questions and 44 conditional questions. The first section concerned dog and family profile (age, postal code, number of people in the family, number of children, profession, age of the dog, neutered status, neutering age, breed, health condition, body condition according to owners, change in weight over time, muscular status). A second section focused on lifestyle (deworming, level of activity according to owners, walk time, sport activity with the dog, habitat of the dog, time spent playing with the dog, presence of other animals and interactions). A third part was about toys and resting places. The fourth part was focused on nutrition (type of diet, amount fed, number of meals, place to buy food, category of the food, composition of the diet). The last part was about the dog’s relationship to their diet (where is presented the diet, how accurate is the amount fed, is another dog present, time to finish the meal, …). A last question was about the authorisation to use data. The questionnaire is present in the supplementary files (See Additional file 1 and Additional file 2).

Survey link was communicated on social media (Facebook, LinkedIn, Instagram), with support by the head of communication of Paris Veterinarian school, Lyon Veterinarian School and Toulouse Veterinarian school. The survey was kept online from the 22^nd^ of April to the 4^th^ of June 2020. No ethics approval and consent to participate was required because of the voluntary and anonymous enrolment.

## Inclusion and exclusion criteria

Owners with dogs aged more than one year and living with the dog were enrolled. To avoid the impact of disease on the dietary choice, dogs with previously diagnosed diseases were excluded (however, conditions like osteoarthritis, dysplasia and ichthyosis reported by the owners were accepted. These conditions are quite common in dogs and do not necessarily involve a change of diet, mostly in case of pathology linked to genetics like dysplasia or ichthyosis). Only one dog per owner was accepted (questions in the survey were used to verify the multiple entries from some owners – postcode, name of dogs, gender, number of dogs, …). Some French speakers from other countries have answered the survey and have been excluded because the postcode was outside France. Questionnaires with missing values regarding family characteristics (region, number of children, age, household, number of dogs, type of diets) were excluded.

## Data transformation and analysis

Data from Google form were transferred into Microsoft Excel. Binary variables were created (living in French metropole area of Paris; neutered, female, gestation, purebred dog, sport activity with the dog, no-gluten food (according the petfood references named by the owner), food reward, measuring food accuracy, walking each day, deworming at least every 6 months, walking more than 6 h/walk without leash, age of 40 years and more). The household place was divided between “French metropole area of Paris” and “Rest of France” because of higher occurrence of the former. The age of owners was split at 40 years to compare with the results by [14] where 39 percent of Raw animal product feeders were 40 years of age or younger in United State population. Owner aging 40 years and older may have different belief in nutritional requirement which should be studied in other survey. The different homemade diets have been grouped as NCD (including commercial BARF). Information about the recipes for NCD was mostly imprecise (lack of information about the amounts of the single ingredients) and did not allow a study of the diet’s nutritional adequacy. The body index reported by owners was not taken into consideration as owners may have used different criteria, not comparable with the standardised approach used during veterinary consultations, in estimating their dogs’ body condition leading to a result of difficult interpretation [23].

Two different population of owners were compared: owners feeding NCD (homemade/commercial BARF, cooked homemade diet, prey model, ….) and owners feeding CD (dry or wet pet food). Owners using both (i.e., kibbles in the morning and cooked homemade food in the evening) were not taken into consideration. Statistical analysis was performed on R (R version 3.5.3) via R Studio (R Studio version 1.1.463). Student’s t-test, chi-squared and Fisher’s test were the statistical tests used for data analysis. A *p*-value < 0.05 was considered statistically significant. Only variables with hypothesis were tested to avoid a multiple test situation.

## Supplementary Information

**Additional file 1.** Questionnaire: Lifestyle and nutrition habits. In this file, the questionnaire used for this study was original written in French and has been translated in English Language.

**Additional file 2.** Original questionnaire: Lifestyle and nutrition habits (French).

## Data Availability

The datasets used and analyzed during the current study are available from the corresponding author on reasonable request.

## Abbreviations

BARF: Biologically Appropriate Raw Food / Bones and Raw Food in United States
NCD: Non-conventional diets
CD: Conventional diets
SD: Standard Deviation
