# Supplementary material for: Comparison of canine owner profile according to food choice: an online preliminary survey in France
Source: BMC Vet Res. 2022 May 4;18:163. doi: 10.1186/s12917-022-03258-9 (PMC9066993; doi:10.1186/s12917-022-03258-9)
Supplement: Supplementary file 1 — Additional file 1. Questionnaire: Lifestyle and nutrition habits. In this file, the questionnaire used for this study was original written in French and has been translated in English Language. [file 12917_2022_3258_MOESM1_ESM.docx]

1. Does this dog living with you ?
   1. Yes
   2. No
2. Did you already filled this questionnaire since january 2020 ?
   1. No
   2. Yes for this dog
   3. Yes for another dog
3. If yes for another dog : Name of the dog : _____________________
4. Postal code : _______________________
5. Number of person in the household (you included) : _________________
6. Number of childs in the household : _____________________
7. Your age :
   1. 18-25 years old
   2. 26-40 years old
   3. 41-60 years old
   4. > 60 years old
8. If appropriate, age of your compagnon :
   1. 18-25 years old
   2. 26-40 years old
   3. 41-60 years old
   4. > 60 years old
9. Your profession
   1. Farmer
   2. Craftmen
   3. Traders
   4. Company managers with 10 or more employees
   5. Liberal and related professions
   6. Itellectual and artistic professions
   7. Company executives
   8. Intermediate professions in education, health, civil services
   9. Intermediary administrative and commercial professions of companies
   10. Technicians
   11. Supervisors and foremen
   12. Public services employes
   13. Company administrative employees
   14. Staff in personal services
   15. Skilled workers
   16. Unskilled workers
   17. Farm worjers
   18. Former farmer
   19. Former craftsmen, traders, buisness leaders
   20. Former executives and intermediate professions
   21. Former employees and workers
   22. Unemployed people who have never worked
   23. Inactive
   24. Vet student, Vet nurses, Veterinarians
   25. Others students
10. If appropriate, the profession of your companion :
    1. Farmer
    2. Craftmen
    3. Traders
    4. Company managers with 10 or more employees
    5. Liberal and related professions
    6. Itellectual and artistic professions
    7. Company executives
    8. Intermediate professions in education, health, civil services
    9. Intermediary administrative and commercial professions of companies
    10. Technicians
    11. Supervisors and foremen
    12. Public services employes
    13. Company administrative employees
    14. Staff in personal services
    15. Skilled workers
    16. Unskilled workers
    17. Farm worjers
    18. Former farmer
    19. Former craftsmen, traders, buisness leaders
    20. Former executives and intermediate professions
    21. Former employees and workers
    22. Unemployed people who have never worked
    23. Inactive
    24. Vet student, Vet nurses, Veterinarians
    25. Others students
11. Name of your dog : _____________________________
12. Birth’s date of your dog : _________________________
13. Your dog is :
    1. A neutered male
    2. A entire male
    3. A neutered female
    4. A entire female
14. If applicable, is your female dog in gestation ?
    1. Yes
    2. Non
15. If yes, since how many days ?
16. Age of the neutering
    1. Before 8 months
    2. Between 8 and 12 months
    3. Between 1 and 2 years
    4. Between 3 and 7 years
    5. After 7 years
    6. I don’t know
17. Is your dog a pure breed ?
    1. Yes
    2. No
18. Breed of the parents : _______________________
19. Breed of the dog : __________________________
20. Inscription on the LOF (Book of French origins)
21. Type of hair
    1. Naked
    2. Short
    3. Mi-long
    4. Long
22. Color the the hair :____________________
23. Muzzle :
    1. Mashed
    2. Normal
    3. Prounouced stop
    4. Non-prounouced stop
24. Chronical disease ?
    1. Yes
    2. Non
25. If yes, which one : _________________________
26. Medication ?
    1. Yes
    2. Non
27. If yes, which ones : ________________________
28. Your dog is
    1. Very thin
    2. Thin
    3. Normal
    4. Large
    5. Very large
29. Can you add a picture of your dog (Top view)
30. You want your dog
    1. Keep thiw weight
    2. Lose weight
    3. Gain weight
31. In the 12 months, how many time do you have weighted your dog ?
    1. None
    2. Once
    3. More than once
32. If applicable, why : __________________________________
33. Place to weight the dog
    1. I do not weight my dog
    2. At home
    3. At the veterinarian clinic
    4. Other
34. Weight change ?
    1. Non
    2. Yes, he has gained weight
    3. Yes, he has lost weight
35. Deworming ?
    1. Yes, each 2 months and more
    2. Yes, each 3 months
    3. Yes, each 6 months
    4. Yes, each years
    5. Sometime
    6. Never
36. Weight of the dog : __________________________________________
37. On a scale of 1 to 10, how active would your dog be? Take into account the game, alone or with you, and its behavior outside if necessary.
38. Do you find your dog to be well muscled?
    1. Yes
    2. No
39. What is the off-leash walking time per week? (outside the confinement period)
    1. Less than 1 hour
    2. 2 to 4h
    3. 4h to 6h
    4. More than 6h
40. What is the leash walking time per week? (outside the confinement period)
    1. Less than 1 hour
    2. 2 to 4h
    3. 4h to 6h
    4. More than 6h
41. Does your dog exercise for sports? If yes which ?
    1. None
    2. Agility
    3. Sled/race
    4. Guide dog
    5. Guard
    6. Hunting
    7. Other
42. If your dog is doing an activity, how many days per week does it represent? (outside the confinement period) : ________________________________
43. On a scale of 1 to 10, how active would you say your dog was ONE YEAR AGO?
44. Type of habitat
    1. Appartement
    2. House
    3. Other
45. What is the interior surface in m² accessible to the dog? ___________________
46. Does the dog have to go up and down stairs?
    1. Yes
    2. No
47. Does your dog have free access to:
    1. Terrace
    2. Kennel
    3. Garden
    4. No accès to outside
48. How often do you take your dog out (outside the garden and home)? (outside the confinement period)
    1. Every day, once
    2. Every day, several times a day
    3. Sometime (i.e, the week-end)
    4. Rarely (i.e, during holiday)
49. If your dog lives part of the year in the city and part in the countryside, what time does he spend, in%, in the countryside? (If he only lives in town, put: 0) :________________
50. How long does the dog go out per day? (outside the confinement period)
    1. Less than 30 min
    2. Between 30 and 60 min
    3. Between 1h and 2 h
    4. More than 2h
51. Do you or a member of your family play with the dog?
    1. Yes, each day, once
    2. Yes, each day, several time a day
    3. Yes, some time
    4. Yes, rarely
    5. Non
52. If you play with your dog, which type of play ? : _________________________________
53. Do you have other animals in contact with your dog?
    1. Yes
    2. No
54. Number of dogs (including the present dog) :________________
55. Number of cats :_________________________
56. Number of rabbits :___________________
57. Nimber of others animals (birds, snake, ferrets, ….) :_________________
58. Interaction of your dogs with your others animals

|  | Each day | Often | Rarely | Sometime | Never |
| --- | --- | --- | --- | --- | --- |
| He plays with it and the game session is accepted by both |  |  |  |  |  |
| He plays with it but the dog is forced |  |  |  |  |  |
| He plays with it but the other animal doesn't want |  |  |  |  |  |
| He sleeps with |  |  |  |  |  |

1. Does your dog share spaces or objects with other pets in the house?
   1. Yes, his water bowls
   2. Yes, his food bowls
   3. Yes, his toys
   4. Yes, his sleeping
   5. Nothing of the sort
2. Is this sharing going well? (Choose the option that corresponds to the most common situation.)
   1. No, my dog ​​refuses the interaction
   2. No, the other animal refuses the interaction
   3. Yes
   4. My dog ​​and my other animals never cross paths
3. Does your dog have toys?
   1. Yes, but he doesn't use it
   2. Yes, and he uses it
   3. No
4. Where does your dog primarily sleep?
   1. Outside in a doghouse or kennel
   2. Inside where he wants
   3. In the garage
   4. Inside in a reserved place (under stairs, ...)
   5. Inside, in the living room
   6. Inside, in the kitchen
   7. Inside, in a room
   8. Other
5. How is his sleep area ?
   1. In height
   2. A carpet
   3. A basket
   4. A sofa
   5. A reserved bed
   6. In your bed
   7. In your child's bed
   8. Other
6. What type of bowl does he have?
   1. A classic bowl
   2. An "anti-glutton" bowl
   3. A distributor
   4. An electronic / automatic bowl
   5. Other
7. Does your dog have toys to split or slow down food intake?
   1. Yes but he does not use it
   2. Yes and he uses it
   3. No
8. What type of food do you primarily feed your dog?
   1. Industrial food (croquettes, boxes or bags)
   2. Home-made ration (BARF, classic home made ration, industrial BARF, Whole prey, ...)
   3. A mixture of both (for example, 50% kibble and 50% homemade ration)

IN CASE OF INDUSTRIAL FOOD :

1. What is your dog's main type of diet?
   1. Complete dry food (croquettes)
   2. Complete food in box or sachet
2. How much do you distribute each day?
   1. An amount prescribed by the veterinarian
   2. A quantity indicated by the food manufacturer (on the back of the bag for example)
   3. A quantity indicated by someone other than the manufacturer or the veterinary team
   4. My dog ​​has unlimited food and eats according to his appetite
3. For croquettes, this quantity is distributed in how many meals?
   1. Self-service (as soon as there is no more food, the dog is served again)
   2. Once a day
   3. Twice a day
   4. More than twice a day
   5. I never give dry food
4. For food in cans or sachets, this quantity is distributed in how many meals?
   1. Self-service (as soon as there is no more food, the dog is served again)
   2. Once a day
   3. Two to four times a day
   4. More than four times a day
   5. I never give wet food
5. Where do you usually buy the main food? (outside the confinement period)
   1. In a garden center or specialized store
   2. In a veterinarian clinic
   3. On the Internet
   4. It depends (on promotions, ...)
   5. Other
6. To which category (ies) does the main food belong? (many possible responses)
   1. Generic food (food for all types of dogs)
   2. Growth or pediatric
   3. Adult
   4. Senior
   5. For netered dog
   6. Light
   7. Dietetic
   8. No grain/no gluten
   9. Vegetarian diet
   10. Primitive
   11. Other
7. In the case of a dietetic food, for which condition is it?
   1. Urinary/renal
   2. Hepatic/pantreatic
   3. Diebetic
   4. Skin or ostheoatrhitis
   5. Other
8. How much kibble do you give per day (if you give it regularly) :___________________
9. Can you send us a photo of the foods you donate with the composition listed?
10. If it is not possible for you to send us a photo of the composition, can you give us the name and the reference of the food?

IN CASE OF HOMEMADE RATION

1. For this ration, how much do you distribute each day?
   1. A prescribed quantity
   2. A free amount according to my dog's appetite
   3. Other
2. How do you split the distribution of this ration?
   1. Once
   2. Two to four times a day
   3. More than four times a day
3. Does the homemade ration belong to one of these categories?
   1. BARF
   2. Whole prey/prey
   3. Vegetarian
   4. No grain (except BARF)
   5. No
4. How do you make the recipe?
   1. Prescribed by a veterinarian in consultation
   2. Prescribed by a veterinarian, online
   3. Personal recipe
   4. Recipe found in a book written by a veterinarian
   5. Recipe found on the internet (facebook group) or in a book but not developed by a veterinarian
5. Does your recipe include food supplements with minerals and vitamins?
   1. Yes
   2. No
6. If yes, which one ? ______________________________
7. Can you give us a precise description of your dog's daily ration? (example: 100 gr of Steak 5%; 1 tablespoon of rapeseed oil ....) :____________________________________
8. If you are using an industrial BARF, can you take a picture of the composition?_____________
9. If it is not possible for you to send us the photo of the industrial BARF feed, can you send us the brand and the reference?___________________________

IN CASE OF MIX INDUSTRIAL+HOMEMADE RATION

1. Please give details of your animal's daily ration (amount of dry food, amount of wet food, amount of table scraps and household food).
2. For this ration, how much do you distribute each day?
   1. A prescribed quantity
   2. A free amount according to my dog's appetite
3. Does the ration belong to one of these categories?
   1. BARF
   2. Whole prey/prey
   3. Vegetarian
   4. No grain (except BARF)
   5. No
4. Can you take a picture of the composition of the processed foods that you feed your pet?____________
5. If it is not possible for you to send us the photo of the food composition, can you give us the reference and the brand?______________________
6. Distribute in addition:
   1. Table scraps, at the table
   2. Table scraps, in the bowl
   3. Nothing of the sort
7. If you are distributing table scraps, can you tell us the approximate composition? (ex: 20 gr of yoghurt base)___________________________________________
8. Do you distribute rewards?
   1. Yes
   2. No
9. If yes, when ?
   1. Education
   2. Work
   3. Play
   4. When you want
10. If yes, which one and which quantity per day ?
11. In which container is his main meal served?
    1. His own
    2. Other
12. Where does the meal take place?
    1. In the kitchen
    2. When the family eat
    3. Alone
    4. On his sleeping place
    5. In his kennel
    6. Outside
    7. Other
13. How many people are likely to feed the dog (usually)?______________________________
14. Are the quantities measured systematically?
    1. Systematic weighing
    2. Rigorous use of a measuring cup (line with the marker for example)
    3. Approximate use of a measuring cup
    4. Estimate according to the duration of the package
    5. No
15. Does your dog eat better in the presence of another dog?
    1. Yes
    2. No
    3. I don’t know
16. If yes, If so, when he is in a group compared to when he is alone does he finish his bowl ...
    1. Faster
    2. Slower
    3. Always the last one
17. Ingestion speed
    1. Normal
    2. Slow
    3. Fast or very fast
18. By submitting this form, I agree that the information entered will be used as part of the study carried out by the National Veterinary Schools of Alfort and Toulouse
    1. Yes
    2. No
