# Supplementary material for: Comparison of canine owner profile according to food choice: an online preliminary survey in France
Source: BMC Vet Res. 2022 May 4;18:163. doi: 10.1186/s12917-022-03258-9 (PMC9066993; doi:10.1186/s12917-022-03258-9)
Supplement: Supplementary file 2 — Additional file 2. Original questionnaire: Lifestyle and nutrition habits (French). [file 12917_2022_3258_MOESM2_ESM.docx]

1- Est-ce que ce chien vit avec vous ?

1. Oui
2. Non

2- Avez-vous déjà rempli ce questionnaire depuis janvier 2020 ?

1. Non
2. Oui pour ce chien
3. Oui pour un autre chien

3- Si oui pour un autre chien : Nom du chien : _____________________

4- Code postal : _______________________

5- Nombre de personne dans le ménage (vous inclus) : _________________

6- Nombre d'enfants dans le ménage : _____________________

7- Votre âge :

1. 18-25 ans
2. 26-40 ans
3. 41-60 ans
4. > 60 ans

8- Le cas échéant, âge de votre compagnon :

1. 18-25 ans
2. 26-40 ans
3. 41-60 ans
4. > 60 ans

9- Votre métier

1. Agriculteurs
2. Artisans
3. Les commerçants
4. Chefs d'entreprise de 10 salariés ou plus
5. Professions libérales et assimilées
6. Professions intellectuelles et artistiques
7. Dirigeants d'entreprise
8. Professions intermédiaires de l'éducation, de la santé, de la fonction publique
9. Professions intermédiaires administratives et commerciales des entreprises
10. Techniciens
11. Surveillants et contremaîtres
12. Employés des services publics
13. Employés administratifs de l'entreprise
14. Personnel des services à la personne
15. Travailleurs qualifiés
16. Travailleurs non qualifiés
17. Travailleurs de la ferme
18. Ancien agriculteur
19. Anciens artisans, commerçants, chefs d'entreprise
20. Anciens cadres et professions intermédiaires
21. Anciens employés et ouvriers
22. Les chômeurs qui n'ont jamais travaillé
23. Inactif
24. Étudiant vétérinaire, Infirmières vétérinaires, Vétérinaires
25. Autres étudiants

10- Le cas échéant, la profession de votre accompagnateur :

1. Agriculteurs
2. Artisans
3. Les commerçants
4. Chefs d'entreprise de 10 salariés ou plus
5. Professions libérales et assimilées
6. Professions intellectuelles et artistiques
7. Dirigeants d'entreprise
8. Professions intermédiaires de l'éducation, de la santé, de la fonction publique
9. Professions intermédiaires administratives et commerciales des entreprises
10. Techniciens
11. Surveillants et contremaîtres
12. Employés des services publics
13. Employés administratifs de l'entreprise
14. Personnel des services à la personne
15. Travailleurs qualifiés
16. Travailleurs non qualifiés
17. Travailleurs de la ferme
18. Ancien agriculteur
19. Anciens artisans, commerçants, chefs d'entreprise
20. Anciens cadres et professions intermédiaires
21. Anciens employés et ouvriers
22. Les chômeurs qui n'ont jamais travaillé
23. Inactif
24. Étudiant vétérinaire, Infirmières vétérinaires, Vétérinaires
25. Autres étudiants

11- Nom de votre chien : _____________________________

12- Date de naissance de votre chien : _________________________

13- Votre chien est :

1. Un mâle stérilisé
2. Un mâle entier
3. Une femelle stérilisée
4. Une femelle entière

14- Le cas échéant, votre chienne est-elle en gestation ?

1. Oui
2. Non

15- Si oui, depuis combien de jours ?

16- Age de la castration

1. Avant 8 mois
2. Entre 8 et 12 mois
3. Entre 1 et 2 ans
4. Entre 3 et 7 ans
5. Après 7 ans
6. Je ne sais pas

17- Votre chien est-il de race pure ?

1. Oui
2. Non

18- Race des parents : _______________________

19- Race du chien : __________________________

20- Inscription au LOF (Livre des Origines Françaises)

21- Type de poils

1. Nu
2. Court
3. Mi-long
4. Long

22- Couleur de la robe :____________________

23- Museau :

1. Ecrasé
2. Normal
3. Stop marqué
4. Stop non marqué

24- Maladie chronique ?

1. Oui
2. Non

25- Si oui, laquelle : _________________________

26- Médicaments ?

1. Oui
2. Non

27- Si oui, lesquels : ________________________

28- Votre chien est

1. Très maigre
2. Un peu maigre
3. Normal
4. Un peu gros
5. Très gros

29- Pouvez-vous ajouter une photo de votre chien (Vue de dessus)

30- Vous voulez votre chien

1. Garde ce poids
2. Maigrisse
3. Grossisse

31- Dans les douze derniers mois, combien de fois votre chien a-t-il été pesé ?

1. Aucune
2. Une fois
3. Plusieurs fois

32- Le cas échéant, pourquoi : __________________________________

33- Où pesez-vous cotre chien le plus souvent

1. Je ne le pèse pas
2. Chez moi
3. A la clinique vétérinaire
4. Autre

34- Le poids de votre chien a-t-il changé dernièrement ?

1. Non je ne pense pas
2. Oui, il a pris du poids
3. Oui, il a maigri

35- Votre chien est-il vermifugé régulièrement

1. Oui, tous les 2 mois
2. Oui, tous les 3 mois
3. Oui, tous les 6 mois
4. Oui, chaque année
5. Occasionnellement
6. Jamais

36- Poids estimé du chien : __________________________________________

37- Sur une échelle de 1 à 10, à combien estimez-vous le degré d’activité de votre chien ? (10 : chien très actif et très sportif) Prenez en compte le jeu, seul ou avec vous, et son comportement à l’extérieur le cas échéant)

38- Trouvez-vous votre chien bien musclé ?

1. Oui
2. Non

39- Quel est le temps de promenade sans laisse par semaine ? (Hors période de confinement)

1. Moins d'1 heure
2. 2 à 4h
3. 4h à 6h
4. Plus de 6h

40- Quel est le temps de promenade en laisse par semaine ? (Hors période de confinement)

1. Moins d'1 heure
2. 2 à 4h
3. 4h à 6h
4. Plus de 6h

41- Votre chien fait-il de m’exercice sportif ? Si oui lequel ?

1. Rien
2. Agility
3. Traîneau/course
4. Chien d'aveugle
5. Garde
6. Chasse
7. Autre

42- Si votre chien fait une activité, combien de jours par semaine cela représente-t-il ? (Hors période de confinement) : ________________________________

43- Et à combien estimez-vous le degré d’activité de votre chien (de 1 à 10),il y a un an ?

44- Type d'habitat

1. Appartement
2. Maison
3. Autre

45- Quelle est la surface intérieure en m² accessible au chien ? ___________________

46- Le chien doit-il monter et descendre des escaliers ?

1. Oui
2. Non

47- Votre chien a-t-il accès gratuitement à :

1. Terrasse
2. Chenil
3. Jardin
4. Pas d'accès à l'extérieur

48- A quelle fréquence sortez-vous votre chien (hors du jardin et de la maison) ? (Hors période de confinement)

1. Chaque jour, une fois
2. Tous les jours, plusieurs fois par jour
3. Parfois (c'est-à-dire le week-end)
4. Rarement (c'est-à-dire pendant les vacances)

49- Si votre chien vit une partie de l'année en ville et une partie à la campagne, combien de temps passe-t-il, en %, à la campagne ? (S'il n'habite qu'en ville, mettez : 0) :________________

50- Combien de temps le chien sort-il par jour ? (hors période de confinement)

1. Moins de 30 minutes
2. Entre 30 et 60 minutes
3. Entre 1h et 2h
4. Plus de 2h

51- Est-ce que vous ou un membre de votre famille jouez avec le chien ?

1. Oui, chaque jour, une fois
2. Oui, chaque jour, plusieurs fois par jour
3. Oui parfois
4. Oui, rarement
5. Non

52- Si vous jouez avec votre chien, quel type de jeu ? : _________________________________

53- Avez-vous d'autres animaux en contact avec votre chien ?

1. Oui
2. Non

54- Nombre de chiens (incluant le chien actuel) :________________

55- Nombre de chats :_________________________

56- Nombre de lapins :___________________

57- Nombre d'autres animaux (oiseaux, serpent, furets, ….) :_________________

58- Interaction de vos chiens avec vos autres animaux

|  | Tout les jours | Souvent | Rarement | Exceptionnellement | Jamais |
| --- | --- | --- | --- | --- | --- |
| Il joue avec et la session de jeu est acceptée par les deux |  |  |  |  |  |
| Il joue avec mais le chien est forcé |  |  |  |  |  |
| Il joue avec mais l’autre animal n’est pas volontaire |  |  |  |  |  |
| Il dort avec |  |  |  |  |  |

59- Votre chien partage-t-il des espaces ou des objets avec d'autres animaux domestiques dans la maison ?

1. Oui, ses bols d'eau
2. Oui, ses bols de nourriture
3. Oui, ses jouets
4. Oui, son sommeil
5. Rien de la sorte

60- Est-ce que ce partage se passe bien ? (Choisissez l'option qui correspond à la situation la plus courante.)

1. Non, mon chien refuse l'interaction
2. Non, l'autre animal refuse l'interaction
3. Oui
4. Mon chien et mes autres animaux ne se croisent jamais

61- Votre chien a-t-il des jouets ?

1. Oui, mais il ne l'utilise pas
2. Oui, et il l'utilise
3. Non

62- Où votre chien dort-il principalement ?

1. A l'extérieur dans une niche ou un chenil
2. A l'intérieur où il veut
3. Dans le garage
4. A l'intérieur dans un endroit réservé (sous escalier, ...)
5. A l'intérieur, dans le salon
6. A l'intérieur, dans la cuisine
7. A l'intérieur, dans une chambre
8. Autre

63- Comment est son coin sommeil ?

1. En hauteur
2. Un tapis
3. Un panier
4. Un canapé
5. Un lit réservé
6. Dans mon lit
7. Dans le lit de mon enfant
8. Autre

64- Quel type de bol a-t-il ?

1. Un bol classique
2. Un bol "anti-glouton"
3. Un distributeur
4. Une gamelle électronique/automatique
5. Autre

65- Votre chien a-t-il des jouets pour diviser ou ralentir la prise de nourriture ?

1. Oui mais il ne l'utilise pas
2. Oui et il l'utilise
3. Non

66- Quel type de nourriture donnez-vous principalement à votre chien ?

1. Alimentaire industriel (croquettes, boites ou sachets)
2. Ration maison (BARF, ration maison classique, BARF industriel, Whole prey, ...)
3. Un mélange des deux (par exemple 50% croquettes et 50% ration maison)

EN CAS D'ALIMENTATION INDUSTRIELLE :

67- Quelle est la principale alimentation de votre chien ?

1. Croquettes complètes (croquettes)
2. Aliment complet en boite ou sachet

68- Combien distribuez-vous chaque jour ?

1. Une quantité prescrite par le vétérinaire
2. Une quantité indiquée par le fabricant de produits alimentaires (au dos du sac par exemple)
3. Une quantité indiquée par quelqu'un d'autre que le fabricant ou l'équipe vétérinaire
4. Mon chien a de la nourriture à volonté et mange selon son appétit

69- Pour les croquettes, cette quantité est répartie en combien de repas ?

1. Libre-service (dès qu'il n'y a plus de nourriture, le chien est à nouveau servi)
2. Une fois par jour
3. Deux fois par jour
4. Plus de deux fois par jour
5. Je ne donne jamais de nourriture sèche

70- Pour les aliments en conserves ou en sachets, cette quantité est répartie en combien de repas ?

1. Libre-service (dès qu'il n'y a plus de nourriture, le chien est à nouveau servi)
2. Une fois par jour
3. Deux à quatre fois par jour
4. Plus de quatre fois par jour
5. Je ne donne jamais de nourriture humide

71- Où achetez-vous habituellement la nourriture principale ? (hors période de confinement)

1. En jardinerie ou magasin spécialisé
2. Dans une clinique vétérinaire
3. Sur Internet
4. Cela dépend (des promotions, ...)
5. Autre

72- A quelle(s) catégorie(s) appartient l'aliment principal ? (plusieurs réponses possibles)

1. Nourriture générique (nourriture pour tous les types de chiens)
2. Croissance ou pédiatrie
3. Adulte
4. Sénior
5. Pour chien stérilisé
6. Light
7. Diététique
8. Sans céréales/sans gluten
9. Régime végétarien
10. Primitif
11. Autre

73- Dans le cas d'un aliment diététique, pour quelle condition s'agit-il ?

1. Urinaire/rénal
2. Hépatique/pancréatique
3. Diabétique
4. Peau ou arthrose
5. Autre

74- Combien de croquettes donnez-vous par jour (si vous en donnez régulièrement) : ___________________

75- Pouvez-vous nous envoyer une photo des aliments que vous donnez avec la composition indiquée ?

76- S'il ne vous est pas possible de nous envoyer une photo de la composition, pouvez-vous nous donner le nom et la référence de l'aliment ?

EN CAS DE RATION MAISON

77- Pour cette ration, combien distribuez-vous chaque jour ?

1. Une quantité prescrite
2. Un montant gratuit selon l'appétit de mon chien
3. Autre

78- Comment répartissez-vous la distribution de cette ration ?

1. Une fois
2. Deux à quatre fois par jour
3. Plus de quatre fois par jour

79- La ration faite maison appartient-elle à l'une de ces catégories ?

1. BAFR
2. Proie entière/proie
3. Végétarien
4. Sans céréales (sauf BARF)
5. Non

80- Comment faites-vous la recette ?

1. Prescrit par un vétérinaire en consultation
2. Prescrit par un vétérinaire, en ligne
3. Recette personnelle
4. Recette trouvée dans un livre écrit par un vétérinaire
5. Recette trouvée sur internet (groupe facebook) ou dans un livre mais non élaborée par un vétérinaire

81- Votre recette comprend-elle des compléments alimentaires avec des minéraux et des vitamines ?

1. Oui
2. Non

82- Si oui, lequel ? ______________________________

83- Pouvez-vous nous décrire précisément la ration journalière de votre chien ? (Exemple : 100 gr de Steak 5% ; 1 cuillère à soupe d'huile de colza....) :____________________________________

84- Si vous utilisez un BARF industriel, pouvez-vous prendre une photo de la composition ?_____________

85- S'il ne vous est pas possible de nous envoyer la photo de l'alimentation industrielle BARF, pouvez-vous nous envoyer la marque et la référence ?___________________________

EN CAS DE MIX RATION INDUSTRIELLE+MAISON

86- Veuillez détailler la ration journalière de votre animal (quantité de nourriture sèche, quantité de nourriture humide, quantité de restes de table et nourriture ménagère).

87- Pour cette ration, combien distribuez-vous chaque jour ?

1. Une quantité prescrite
2. Un montant gratuit selon l'appétit de mon chien

88- La ration appartient-elle à l'une de ces catégories ?

1. BAFR
2. Proie entière/proie
3. Végétarien
4. Sans céréales (sauf BARF)
5. Non

89- Pouvez-vous prendre une photo de la composition des aliments transformés que vous donnez à votre animal ?____________

90- S'il ne vous est pas possible de nous envoyer la photo de la composition de l'aliment, pouvez-vous nous donner la référence et la marque ?______________________

91- Distribuer en plus :

1. Restes de table, à table
2. Restes de table, dans le bol
3. Rien de la sorte

92- Si vous distribuez des restes de table, pouvez-vous nous en dire la composition approximative ? (ex : 20 gr de base de yaourt)________________________________________________________

93- Distribuez-vous des récompenses ?

1. Oui
2. Non

94- Si oui, quand ?

1. Éducation
2. Travail
3. Jouer
4. Quand tu veux

95- Si oui, lequel et quelle quantité par jour ?

96- Dans quel récipient est servi son repas principal ?

1. Son propre
2. Autre

97- Où se déroule le repas ?

1. Dans la cuisine
2. Quand la famille mange
3. Seul
4. Sur son lieu de couchage
5. Dans son chenil
6. Dehors
7. Autre

98- Combien de personnes sont susceptibles de nourrir le chien (habituellement) ?______________________________

99- Les quantités sont-elles mesurées systématiquement ?

1. Pesée systématique
2. Utilisation rigoureuse d'un gobelet doseur (ligne avec le marqueur par exemple)
3. Utilisation approximative d'une tasse à mesurer
4. Estimation selon la durée du forfait
5. Non

100- Votre chien mange-t-il mieux en présence d'un autre chien ?

1. Oui
2. Non
3. Je ne sais pas

101- Si oui, lorsqu'il est en groupe par rapport à quand il est seul finit-il sa gamelle...

1. Plutôt plus vite
2. Plutôt moins vite
3. Systématiquement le dernier

102- Vitesse d'ingestion

1. Normale
2. Lente
3. Rapide ou très rapide

103- En soumettant ce formulaire, j'accepte que les informations saisies soient exploitées dans le cadre de l'étude menée par les Ecoles Nationales Vétérinaires d'Alfort et de Toulouse

1. Oui
2. Non
